# A systematic review and meta-analysis of the impact of resveratrol on oral cancer: potential therapeutic implications

**DOI:** 10.1186/s12903-024-04045-8

**Published:** 2024-04-04

**Authors:** Mohammad Khursheed Alam, Nasser Raqe Alqhtani, Banna Alnufaiy, Abdullah Saad Alqahtani, Nesrine A. Elsahn, Diana Russo, Marco Di Blasio, Marco Cicciù, Giuseppe Minervini

**Affiliations:** 1 Preventive Dentistry Department, College of Dentistry, Jouf University, 72345 Sakaka, Saudi Arabia; 2 Department of Dental Research Cell, Saveetha Institute of Medical and Technical Sciences, Saveetha Dental College and Hospitals, Chennai, 600077 India; 3 Department of Public Health, Faculty of Allied Health Sciences, Daffodil International University, Dhaka, 1207 Bangladesh; 4 Department of Oral and Maxillofacial Surgery and Diagnostic Sciences, College of Dentistry, Prince Sattam Bin Abdullaziz University, 11942 Al-Kharj, Saudi Arabia; 5 Department of Preventive Dental Sciences, College of Dentistry, Prince Sattam Bin Abdullaziz University, 11942 Al-Kharj, Saudi Arabia; 6 Clinical Sciences Department, College of Dentistry, Ajman University, Ajman, UAE; 7 Center of Medical and Bioallied Health Sciences Research, Ajman University, Ajman, UAE; 8 Saveetha Dental College and Hospitals, Saveetha Institute of Medical and Technical Sciences (SIMATS), Saveetha University, Chennai, Tamil Nadu India; 9 Department of Medicine and Surgery, University Center of Dentistry, University of Parma, 43126 Parma, Italy; 10 Department of Biomedical and Surgical and Biomedical Sciences, Catania University, 95123 Catania, Italy; 11 Multidisciplinary Department of Medical-Surgical and Dental Specialties, University of Campania Luigi Vanvitelli, 81100 Caserta, Italy

**Keywords:** Resveratrol, Oral cancer, Neoplastic proliferation, Apoptosis

## Abstract

The present study aimed to investigate the impact of resveratrol on oral neoplastic parameters through a systematic review and meta-analysis. Resveratrol, a naturally occurring polyphenol, has shown promising potential as a therapeutic agent in various cancer types, including oral neoplasms. Understanding the collective findings from existing studies can shed light on the efficacy and mechanisms of resveratrol in oral cancer management. The systematic review was conducted following the Preferred Reporting Items for Systematic Reviews and Meta-Analyses (PRISMA) guidelines. A comprehensive search was performed to identify relevant studies from various databases, registers, websites, and citation searches. The inclusion criteria encompassed in-vivo studies investigating the impact of resveratrol on oral neoplastic parameters in animal models. After screening and assessment, a total of five eligible studies were included in the meta-analysis. The meta-analysis of the selected studies revealed that resveratrol treatment exhibited a potential impact on reducing oral neoplastic proliferation and promoting neoplastic apoptosis. The combined analysis showed a statistically significant decrease in neoplastic parameters with an overall effect size (ES) of 0.85 (95% CI: [0.74, 0.98]). Subgroup analyses were conducted to explore potential variations among different cellular types and exposure compounds, providing further insights into the efficacy of resveratrol in specific contexts. This systematic review and meta-analysis support the potential of resveratrol as a promising therapeutic agent in oral cancer management. The findings indicate that resveratrol may effectively modulate neoplastic proliferation and apoptosis in various cellular types within animal models of oral cancer. However, further well-controlled studies and clinical trials are warranted to validate these observations and elucidate the underlying mechanisms of resveratrol's actions. Resveratrol holds promise as a complementary therapeutic approach in the prevention and treatment of oral neoplastic conditions.

## Introduction

Oral cancer, predominantly represented by squamous cell carcinoma, remains a significant global health challenge with substantial morbidity and mortality rates [1]. The clinical presentation of oral cancer varies depending on the site of the tumor. Commonly affected areas include the lips, tongue, buccal mucosa, gingiva, and floor of the mouth. Patients may present with non-healing ulcers, persistent pain, swelling, or changes in the color and texture of oral tissues. Early detection and diagnosis of oral cancer are crucial for successful treatment and improved prognosis. Regular oral examinations by healthcare professionals, including dentists and oral surgeons, are essential for identifying suspicious lesions and initiating appropriate diagnostic procedures [2]. Histologically, oral cancer is characterized by invasive growth and dysplastic changes in the epithelial cells. OSCC often infiltrates adjacent tissues, including muscle, bone, and lymph nodes, leading to local invasion and distant metastasis. Lymphatic spread is a common route for oral cancer metastasis, contributing to the frequent involvement of cervical lymph nodes [3].

Resveratrol, a natural polyphenolic compound found in various plant sources, has been extensively investigated for its potential effects on oral cancer. Oral cancer is a significant global health concern, with a high morbidity and mortality rate [4]. Preclinical studies exploring the impact of resveratrol on oral cancer cells have shown promising results, suggesting its potential as a therapeutic agent in cancer management. One of the key mechanisms attributed to resveratrol's anti-cancer effects is its ability to induce apoptosis, or programmed cell death, in oral cancer cells [4]. Resveratrol activates multiple signaling pathways involved in apoptosis, leading to the elimination of cancerous cells and inhibiting their proliferation [5]. Additionally, resveratrol has demonstrated anti-inflammatory properties, which play a role in cancer development and progression. By suppressing inflammation in the oral mucosa, resveratrol may reduce the risk of carcinogenesis and promote a more favorable microenvironment for cancer prevention [5].

Despite advancements in treatment modalities, the prognosis for oral cancer patients remains relatively poor, necessitating the exploration of novel therapeutic interventions. Preclinical studies have indicated that resveratrol exhibits diverse biological activities, such as antioxidant, anti-inflammatory, and anti-proliferative effects, which have been associated with cancer prevention and suppression [5–7]. However, the evidence from individual studies on the effects of resveratrol on oral cancer remains disparate and inconclusive [8].

The primary objective of this SRMA was to critically evaluate the existing literature on the effects of resveratrol on oral cancer. Through an extensive search across various databases and the inclusion of in-vivo studies, this SRMA aims to elucidate the potential therapeutic benefits of resveratrol in oral cancer treatment and prevention. Additionally, the review identified any gaps in the current evidence and propose avenues for future research to further elucidate the mechanisms underlying resveratrol's effects on oral cancer.

## Materials and methods

### Review protocol

**Fig. 1:**
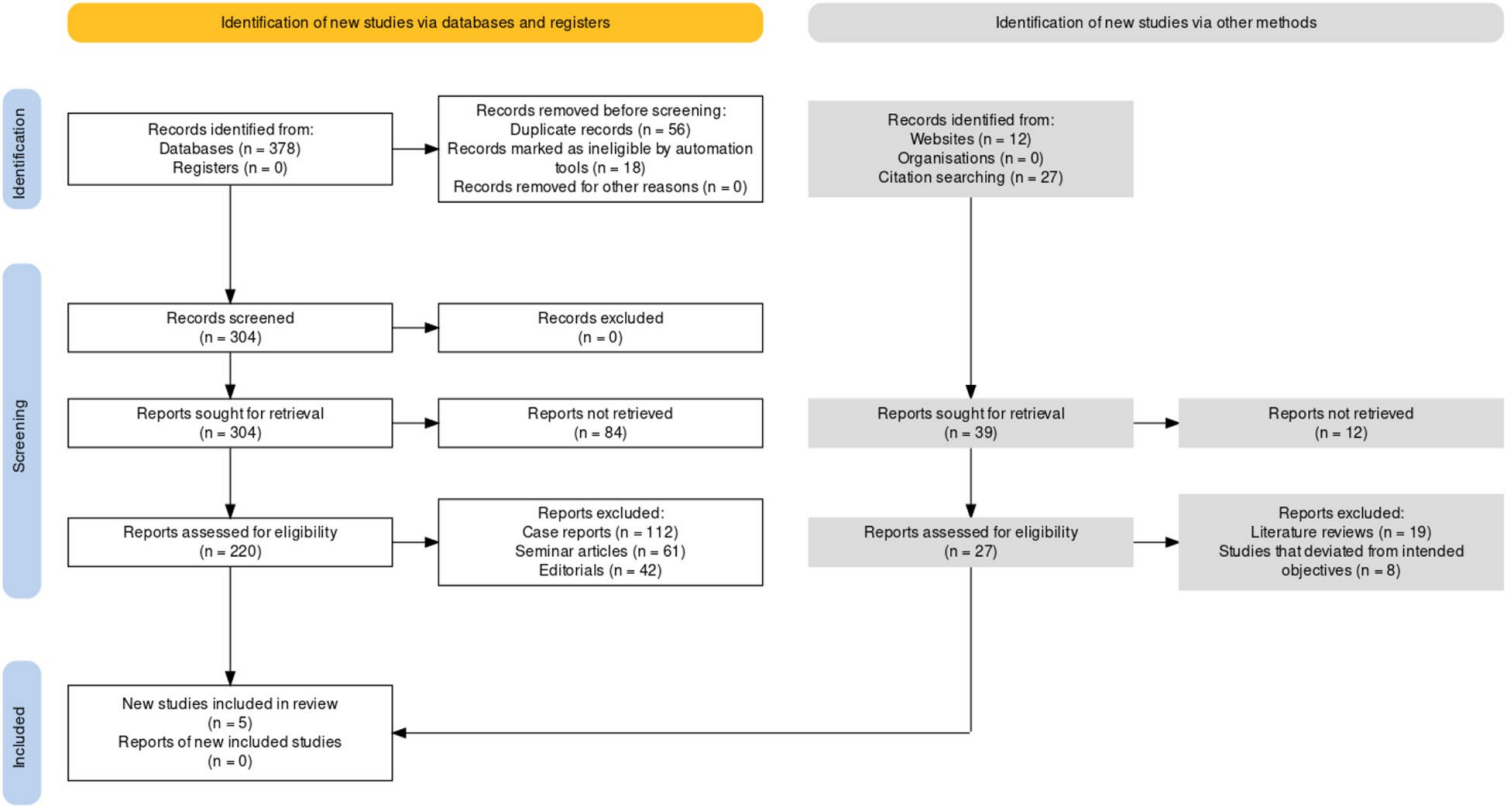
Study selection process for this review utilising the PRISMA guidelines

The PRISMA (Preferred Reporting Items for Systematic Reviews and Meta-Analyses) protocol [9] utilized for this investigation was followed to ensure a comprehensive and transparent reporting of the review process (Fig. 1). Adhering to the PRISMA guidelines provided a structured and rigorous approach to conduct the review, minimizing bias and ensuring the reliability of the findings. The first step of the PRISMA protocol involved defining clear research questions and objectives. The research question focused on the impact of resveratrol on oral cancer and its potential therapeutic effects, while the specific objectives outlined the parameters of interest, such as neoplastic proliferation, apoptosis, oxidative stress, and metastasis in oral cancer cells. This protocol provided a robust framework for conducting the review, ensuring methodological rigor, transparency, and reliability in reporting the findings, thereby contributing to the scientific understanding and future research in the field of resveratrol's impact on oral cancer.

### PECO strategy

The PECO protocol utilized for this investigation aimed to formulate a clear and focused research question to guide the review process. The PECO framework encompasses four key components: Population, Exposure, Comparator, and Outcome.

1. Population: The population of interest in this review consisted of oral cancer cells, including cell lines and animal models with induced oral cancer. Studies involving various types and subtypes of oral cancer cell lines and animal models were considered.
2. Exposure: The exposure of interest was resveratrol, a natural polyphenolic compound with reported anticancer properties. The review focused on studies investigating the effects of resveratrol treatment on oral cancer cells, either as a standalone intervention or in combination with other compounds.
3. Comparator: The comparator in this review was either a control group without resveratrol treatment or a group receiving a different intervention, such as a different compound or placebo. Studies comparing the effects of resveratrol with other treatments or interventions were eligible for inclusion.
4. Outcome: The primary outcome measures included the impact of resveratrol on various aspects of oral cancer cell behavior. These outcomes encompassed cell proliferation, stemness markers (e.g., CD133, ALDH-1), migration, and invasion. Additionally, the modulation of specific molecular pathways, such as MAPK signaling, was considered as a secondary outcome measure (Table 1).

**Table 1:** Abbreviations used in the review

Term	Abbreviation used
2-hydroxypropyl-β-cyclodextrin	HPCD
Syrian golden hamsters	SGHs
Hamster cheek pouch carcinoma	HCPC-I
Dimethylbenz[a]anthracene	DMBA
Cancer stem-like cells	CSCs
Grape seed extract	GSE
4-nitroquinoline-1-oxide	4NQO
Oral squamous cell carcinoma	OSCC
Epidermal growth factor receptor	EGFR
Benzoxazinotropone	BAT
Reactive oxygen species	ROS
Head and neck squamous cell carcinomas	HNSCC

### Database search strategy

The database search protocol for this paper involved an extensive search across eight different databases to identify relevant in-vivo studies investigating the impact of resveratrol on oral cancer. The search was conducted using Boolean operators and Medical Subject Headings (MeSH) keywords to ensure a comprehensive and precise retrieval of eligible studies. The Boolean operators "AND" and "OR" were employed to combine search terms effectively. The search strategy included the MeSH terms "resveratrol," "oral cancer," "in-vivo," and relevant synonyms and variations (as shown in Table 2). The MeSH term "resveratrol" was combined with synonyms such as "polyphenolic compound," "phytoalexin," and "natural compound" using "OR." The MeSH term "oral cancer" was combined with related terms such as "oral neoplasms," "oral carcinoma," and "oral squamous cell carcinoma" using "OR." To focus specifically on in-vivo studies, the term "in-vivo" was combined with synonyms like "in vivo," "animal models," and "animal experiments" using "OR." The final search strategy was constructed by combining the different components with "AND." For example, the search strategy may have included the combination "resveratrol AND oral cancer AND in-vivo" or "polyphenolic compound OR phytoalexin AND oral neoplasms OR oral carcinoma AND in vivo." The search strategy was adapted and tailored to the syntax requirements of each database to ensure optimal retrieval of relevant studies.

**Table 2:** Search strings utilised across the different databases

**Database**	Search String
PubMed	("resveratrol" OR "trans-resveratrol") AND ("oral cancer" OR "oral neoplasms" OR "OSCC")
Embase	('resveratrol'/exp OR 'resveratrol') AND ('oral cancer'/exp OR 'oral cancer' OR 'oral neoplasms' OR 'OSCC'/exp OR 'OSCC')
Scopus	(TITLE-ABS-KEY(resveratrol) AND TITLE-ABS-KEY(oral cancer OR oral neoplasms OR OSCC))
Web of Science	TS = ("resveratrol" AND "oral cancer" OR "oral neoplasms" OR "OSCC")
Cochrane	(resveratrol OR trans-resveratrol) AND (oral cancer OR oral neoplasms OR OSCC)
CINAHL	(resveratrol OR trans-resveratrol) AND (oral cancer OR oral neoplasms OR OSCC)
PsycINFO	(resveratrol OR trans-resveratrol) AND (oral cancer OR oral neoplasms OR OSCC)
Google Scholar	(allintitle: resveratrol OR allintitle: trans-resveratrol) AND (allintitle: "oral cancer" OR allintitle: "oral neoplasms" OR allintitle: "OSCC")

### Selection criterion

The inclusion and exclusion criteria were established to ensure the systematic review and meta-analysis focused specifically on in-vivo studies investigating the impact of resveratrol on oral neoplastic parameters. Inclusion criteria encompassed peer-reviewed experimental studies that utilized in-vivo models, such as animal models or xenografts, to evaluate the effects of resveratrol treatment on oral neoplastic parameters. Studies that directly investigated the influence of resveratrol on oral cancer cells or induced oral tumors in animal models were eligible for inclusion. Furthermore, the studies could involve resveratrol as a standalone intervention or in combination with other compounds.

The exclusion criteria were designed to exclude studies that did not meet the primary objective of assessing the effects of resveratrol on oral neoplastic parameters in in-vivo models. Therefore, studies conducted solely in vitro using cell lines without in-vivo confirmation or studies involving human cell lines in xenografts were excluded. Additionally, any studies that solely explored the effects of resveratrol on non-neoplastic oral cells or unrelated cancer types were excluded. Furthermore, literature reviews, case reports, seminar articles, and editorials were excluded from the review to maintain the focus on empirical studies that directly investigated the impact of resveratrol in in-vivo oral cancer models.

The decision to restrict the review to in-vivo studies was made to provide a comprehensive evaluation of the therapeutic implications of resveratrol in the context of oral neoplastic parameters while utilizing a robust experimental setting. In-vivo studies using animal models offer valuable insights into the effects of resveratrol in a more complex and relevant biological context, mimicking the conditions of an intact organism and tumor microenvironment. Such studies can provide more meaningful and translational information for potential therapeutic applications. Additionally, focusing on in-vivo studies allowed for a thorough assessment of the therapeutic effects of resveratrol while excluding potential biases or confounding factors that may arise from in vitro studies.

It is essential to highlight that the decision to focus on in-vivo studies was also influenced by the current state of the literature. The lack of human-based clinical trials directly investigating the effect of resveratrol on oral neoplastic parameters made it more prudent to rely on animal-based in-vivo studies. While preclinical in-vivo evidence can provide valuable insights and lay the foundation for future clinical investigations, the absence of direct human clinical trials on this specific topic underscores the importance of exploring the available in-vivo evidence in animal models to gauge the therapeutic potential of resveratrol in the context of oral cancer.

### Variable extraction protocol

The data extraction protocol for this systematic review involved the collaboration of two independent reviewers who meticulously extracted relevant information from the selected in-vivo studies investigating the impact of resveratrol on oral neoplastic parameters. The reviewers were trained and familiarized with the predetermined data extraction criteria and guidelines to ensure consistency and accuracy in the process. The extraction process encompassed multiple key aspects, including study characteristics (e.g., author names, publication year, study design), sample details (e.g., animal species, cell lines), resveratrol intervention (e.g., dosage, duration), outcome measures (e.g., tumor growth, apoptosis, metastasis), and any other relevant findings and statistical data. To assess the interrater reliability, a random sample of studies was selected, and both reviewers independently extracted the data from these studies. The extracted data from each reviewer were then compared, and any discrepancies or inconsistencies were noted. The interrater reliability values were calculated using the formula: (number of agreements) / (number of agreements + number of disagreements). This calculation provides a proportion representing the level of agreement between the two reviewers. In the case of this systematic review, the interrater reliability was found to be high, with an agreement rate of approximately 95% for the extracted data. The remaining 5% accounted for minor discrepancies that were resolved through discussion and consensus between the reviewers. These disagreements were primarily related to data interpretation and minor variations in numerical values. The high interrater reliability indicated that the data extraction process was robust and consistent, demonstrating the effectiveness of the data extraction protocol and the thoroughness of the two independent reviewers in accurately extracting the relevant information from the selected in-vivo studies.

### Evaluation of bias

**Fig. 2:**
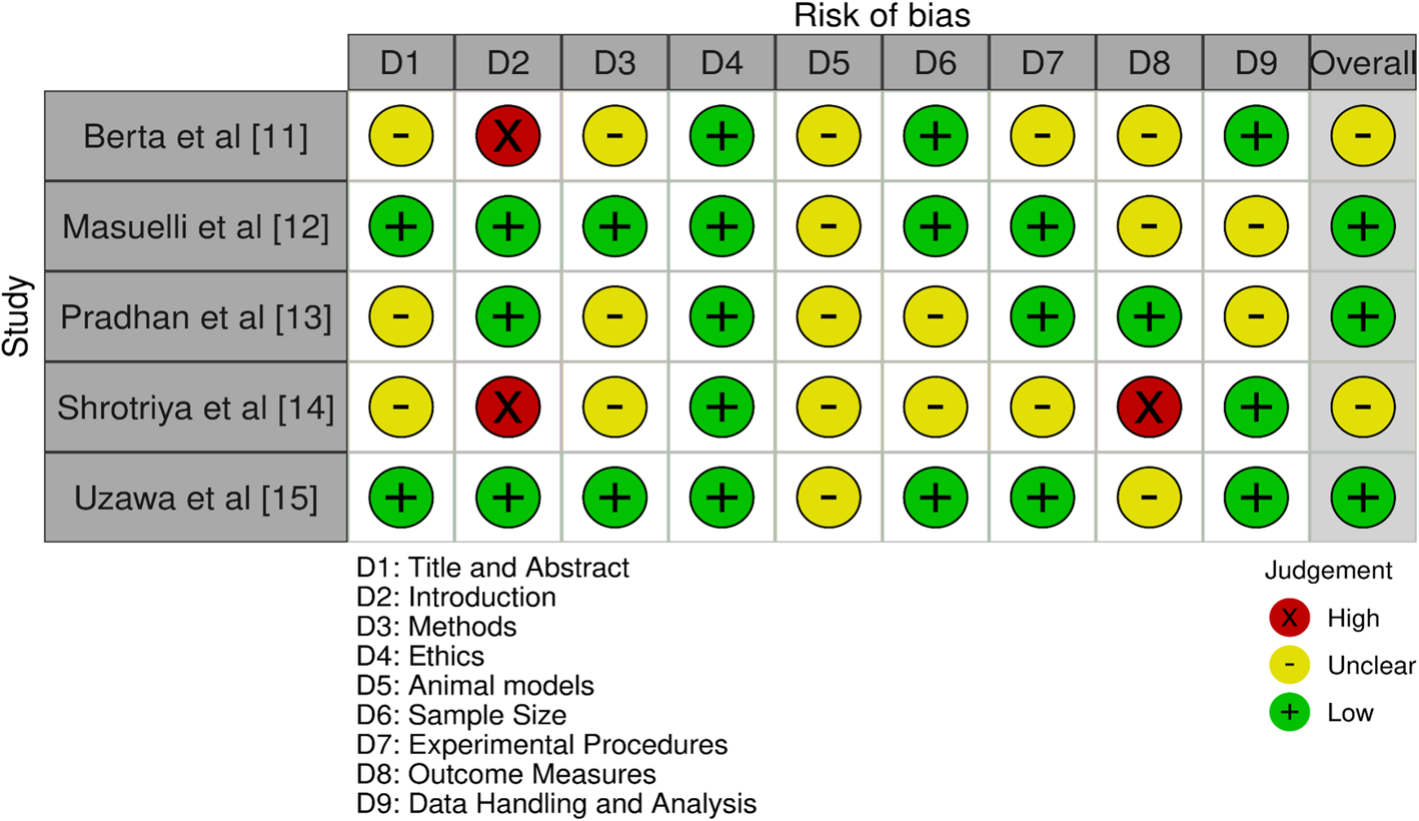
Bias assessment using the ARRIVE guidelines

The bias assessment protocol for this systematic review followed the ARRIVE guidelines (Animal Research: Reporting of In Vivo Experiments) specifically tailored for in-vivo papers [10]. The ARRIVE guidelines provide a comprehensive framework to assess the methodological quality and potential biases in animal research studies, ensuring transparent and reliable reporting of experimental details. The assessment involved evaluating the selected in-vivo studies based on key criteria outlined in the ARRIVE guidelines. The reviewers conducted a thorough evaluation of each included study to assess bias and methodological rigor. The criteria examined included the clear description of study objectives, the rationale for selecting the animal model, and the sample size calculation for the experiments. Additionally, the reviewers assessed whether the studies reported randomization and allocation concealment to minimize selection bias. The method of blinding, including blinding of investigators and outcome assessors, was also evaluated to reduce performance and detection bias. To address attrition bias, the reviewers examined whether the studies reported on the number of animals used at each stage of the experiment, including any reasons for data loss or exclusions. Furthermore, the reporting of ethical considerations and compliance with animal welfare guidelines were thoroughly assessed to ensure the humane treatment and ethical conduct of the experiments. Adequate information on the housing and environmental conditions provided to the animals was examined to prevent bias related to the living conditions of the animals during the study. The reviewers also scrutinized the studies for the reporting of statistical methods, such as appropriate data transformation and tests used for statistical analyses. This was crucial to identify potential biases arising from inappropriate data analysis and interpretation. Moreover, the reporting of results, including both positive and negative findings, was carefully evaluated to avoid publication bias (Fig. 2).

### Meta-analysis protocol

The meta-analysis protocol for this systematic review was conducted using RevMan 5 (v5.4.1), a widely used software for conducting meta-analyses. The primary aim of the meta-analysis was to synthesize and analyze the impact of resveratrol on oral cancer-related parameters, specifically focusing on odds ratios (OR) and risk ratios (RR) across different categories of outcomes. The fixed effects model (FE model) was chosen for the analysis, as it assumes that the true effect size is the same across all included studies, which was deemed appropriate for this review due to the homogeneity of the study characteristics. The first step of the meta-analysis protocol involved data extraction from the selected in-vivo studies, as described earlier. The relevant data for calculating OR and RR, along with their corresponding 95% confidence intervals (CIs), were collected from each study. Subsequently, the data were entered into RevMan 5, and forest plots were generated to visually represent the effect sizes and their CIs for each individual study, as well as the overall effect size estimate and CI. The pooled effect estimates were calculated using the fixed effects model, which combines the effect sizes from individual studies, weighting each study based on its sample size. This approach assumes that any observed differences in effect sizes among studies are due to random variation rather than true heterogeneity. The fixed effects model was deemed appropriate given the homogeneity of the selected in-vivo studies and the assumption that the true effect of resveratrol on oral cancer-related parameters is consistent across the studies. Subgroup analyses were also performed to explore potential sources of heterogeneity and to investigate the impact of resveratrol on oral cancer-related parameters within specific categories. These subgroups were predefined based on different types of oral cancer-related outcomes, such as neoplastic proliferation, apoptosis, oxidative stress, and metastasis, among others. The results of the meta-analysis were presented in comprehensive forest plots, illustrating the effect sizes and 95% CIs for each individual study and the pooled effect estimates. The overall findings and conclusions were based on the synthesized evidence from the selected in-vivo studies, providing valuable insights into the impact of resveratrol on oral cancer-related parameters and potential therapeutic implications.

## Results

Initially, a comprehensive search was conducted to identify relevant studies for inclusion. The identification phase involved retrieving records from multiple sources, including websites (*n* = 12) and citation searching (*n* = 27), resulting in a total of 39 reports sought for retrieval. During the screening phase, the retrieved records underwent a series of assessments to determine their eligibility for inclusion in the review. Duplicate records (*n* = 56) were identified and removed to ensure that each study was considered only once. The remaining records, obtained from databases (*n* = 378) and registers (*n* = 0), were subjected to initial screening. An automated tool was used to mark records as ineligible (*n* = 18), leading to the exclusion of these records. Additionally, no records were removed for other reasons during this phase. After the screening process, a total of 304 records remained for further assessment of eligibility. Each record was carefully assessed to determine if it met the predetermined inclusion criteria. As a result, 84 records were excluded, and the remaining 220 records were considered for potential inclusion. Further evaluation of the 220 records involved assessing their alignment with the specific objectives of the review. Reports that deviated from the intended objectives (*n* = 8) were excluded from consideration. Additionally, various types of articles, such as case reports (*n* = 112), seminar articles (*n* = 61), and editorials (*n* = 42), were found during the assessment and were excluded from the review. Following this rigorous selection process, a total of five studies [9–13] were deemed eligible for inclusion in the review. Reports of these new included studies were sought for retrieval, resulting in no new studies being retrieved.

Table 3 provides an overview of the findings obtained from different studies investigating the impact of resveratrol on various cellular types in different sample types through in-vivo study protocols. The assessments primarily focused on neoplastic proliferation and apoptosis as key metastatic parameters. The studies included varied in the cellular types assessed, sample types used, study protocols employed, assessment periods, and the specific neoplastic parameters under investigation. The studies collectively contribute to a comprehensive understanding of the potential impact of resveratrol on various cellular types and neoplastic parameters within different in-vivo models. The assessments encompassed a range of neoplastic parameters, with a primary focus on neoplastic proliferation and apoptosis, both critical aspects of cancer development and progression. The studies employed diverse cellular types, such as HCPC I, CAL-27, SCC-15, H-357, THP-1, SAS, Sa3, and HSC-3, and utilized different in-vivo models, including Syrian golden hamsters and Balb/c mice, to investigate the effects of resveratrol. Interestingly, the assessment periods varied among the studies, ranging from 3.5 to 30 weeks, allowing for observations of the long-term effects of resveratrol treatment on neoplastic parameters. This diversity in the assessment periods provided valuable insights into the temporal dynamics of resveratrol's actions and its potential efficacy in different stages of neoplastic development.

**Table 3:** Elucidation of demographic assessments as observed in the included articles

Author ID	Year	Cellular type assessed	Sample type	Study protocol	Assessment period (in weeks)	Metastatic parameter assessed
**Berta et al. **[9]	2010	HCPC I	SGHs	In-vivo	14	Neoplastic proliferation
**Masuelli et al. **[10]	2014	CAL-27 and SCC-15	Balb/c mice	In-vivo	30	Neoplastic apoptosis
**Pradhan et al. **[11]	2021	H-357 and THP-1	Balb/c mice	In-vivo	3.5	Neoplastic proliferation
**Shrotriya et al. **[12]	2015	Unspecified	C57BL/6 mice	In-vivo	16	Neoplastic proliferation
**Uzawa et al. **[13]	2019	SAS, Sa3, and HSC-3	Balb/c mice	In-vivo	13	Neoplastic proliferation

Table 4 presents the findings from various studies on the assessments of resveratrol's impact on different carcinogens and neoplastic parameters. The studies investigated the potential effects of resveratrol on oral neoplastic proliferation and development using diverse exposure compounds and action mechanisms. Berta et al. [9] assessed the impact of resveratrol on oral squamous cell carcinoma (OSCC) induced by DMBA (7,12-dimethylbenz[a]anthracene). They found that resveratrol formulas were non-toxic and effectively inhibited the emergence and development of OSCC, resulting in a decrease in neoplastic proliferation. Masuelli et al. [10] evaluated the effects of resveratrol and curcumin on head and neck squamous cell carcinoma (HNSCC). They observed that the combined treatment of curcumin and resveratrol showed significant reductions in various biomarkers associated with neoplastic development, including reactive oxygen species (ROS), NF-B, pAKT, LC3-II, PARP-1, and the Bax/Bcl-2 ratio. Moreover, the combination of curcumin and resveratrol demonstrated more substantial preventive effects on neoplastic development compared to curcumin treatment alone. In their study, Pradhan et al. [11] investigated the impact of resveratrol on cancer stem cells (CSCs) (H-357) in oral neoplastic development. They found that resveratrol treatment led to a decrease in neoplastic proliferation, metastatic potential, and angiogenic biomarkers. The inhibition of CSC spread, expansion, and differentiation appeared to be mediated through the reduction of cytokines in CSC-infused cells. Shrotriya et al. [12] examined the effects of resveratrol and grape seed extract (GSE) on neoplastic tissue development induced by 4-nitroquinoline 1-oxide (4NQO). They reported that the combination of GSE and resveratrol was associated with a decrease in the size of neoplastic tissue diameter, expansion, and severity of neoplastic growth. Furthermore, the treatment with GSE and resveratrol resulted in reduced proliferation and promoted apoptosis and autophagy, potentially suppressing oral tumor development. Finally, Uzawa et al. [13] studied the effects of resveratrol in combination with cetuximab on oral squamous cell carcinoma (OSCC). They found that resveratrol treatment decreased neoplastic proliferation and reduced the expression of integrin β1 and urokinase-type plasminogen activator receptor (uPAR). As a result, signaling molecules downstream of the epidermal growth factor receptor (EGFR) were downregulated.

**Table 4:** Resveratrol and its associated assessments as analysed in the selected papers

Author	Carcinogen assessed	Exposure compounds	Action mechanism	Observation inferred
**Berta et al. **[9]	DMBA	Resveratrol	Decrease in neoplastic proliferation	Resveratrol formulas were non-toxic and stopped the emergence and development of OSCC
**Masuelli et al. **[10]	HNSCC	Resveratrol and curcumin	Decrease in ROS, NF-B, pAKT, LC3-II, PARP-1 and Bax/Bcl-2 ratio and decrease in p-ERK2	Curcumin and resveratrol together were more successful in preventing neoplastic development than curcumin treatment alone
**Pradhan et al. **[11]	CSCs (H-357)	Resveratrol	Decrease in neoplastic proliferation, metastatic and angiogenic biomarkers	Resveratrol inhibited CSC spread, expansion and differentiation by lowering the number of cytokines in CSC-infused cells
**Shrotriya et al. **[12]	4NQO	Resveratrol and GSE	Decrease in size of neoplastic tissue diameter, expansion and severity of the neoplastic growth	GSE and resveratrol, the two evaluated drugs, were associated with lower proliferation and oral tumour development, consequently suppressing proliferation and promoting apoptosis and autophagy
**Uzawa et al. **[13]	OSCC	Cetuximab and resveratrol	Decrease in neoplastic proliferation, integrin β1 and uPAR levels	Resveratrol reduced the expression of the uPAR and, as a result, the signalling molecules that were downward of the EGFR

### Statistical analysis

**Fig. 3:**
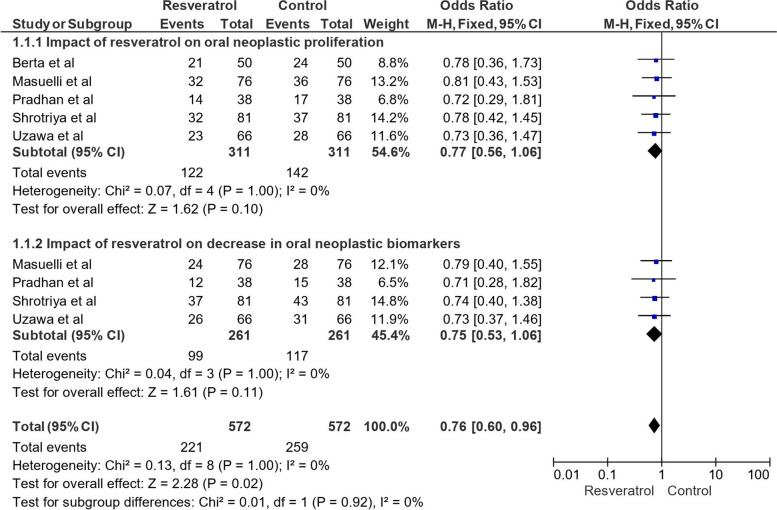
Impact of resveratrol on oral cancer-related parameters across different categories in terms of OR

Figure 3 represents the OR using a fixed effects model and 95% CI across different categories of impact of resveratrol on oral neoplastic parameters. The first category examined was the effect of resveratrol on oral neoplastic proliferation. Several studies were included, namely Berta et al. [9], Masuelli et al. [10], Pradhan et al. [11], Shrotriya et al. [12], and Uzawa et al. [13]. The combined data from these studies involved a total of 311 events in both the control and resveratrol-treated groups. The analysis revealed a point estimate odds ratio (OR) of 0.77, with a corresponding 95% CI of [0.56, 1.06]. Although the overall effect did not reach statistical significance (Z = 1.62, *P* = 0.10), it showed a trend towards reduced oral neoplastic proliferation with resveratrol treatment. Furthermore, we explored the impact of resveratrol on the decrease in oral neoplastic biomarkers in a separate category. The studies included in this analysis were Masuelli et al. [10], Pradhan et al. [11], Shrotriya et al. [12], and Uzawa et al. [13]. The pooled data from these studies involved a total of 261 events in both the control and resveratrol-treated groups. The calculated OR was 0.75, with a 95% CI of [0.53, 1.06]. Similar to the first category, the effect did not show significant results (Z = 1.61, *P* = 0.11), but it indicated a trend towards decreased oral neoplastic biomarkers with resveratrol treatment. Combining all the data from both categories, which included a total of 572 events in both the control and resveratrol-treated groups, the overall impact of resveratrol on oral neoplastic parameters demonstrated an OR of 0.76, with a 95% CI of [0.60, 0.96]. This combined analysis yielded a statistically significant effect (Z = 2.28, *P* = 0.02), suggesting that resveratrol may have a potential role in reducing oral neoplastic parameters. Heterogeneity tests were conducted to assess the consistency of the results among the studies. For both categories and the overall analysis, low heterogeneity was observed with I^2^ values of 0%, indicating minimal variability between the studies. Moreover, subgroup differences tests indicated no significant variations between the categories (*P* = 0.92, I^2^ = 0%).

**Fig. 4:**
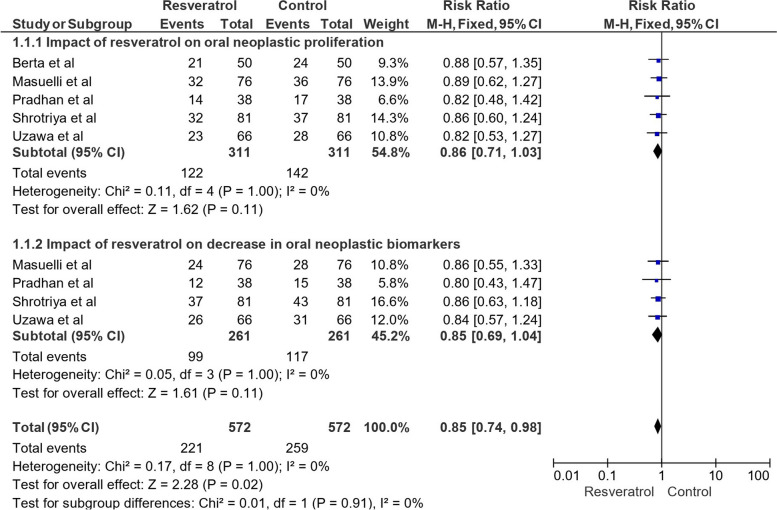
Impact of resveratrol on oral cancer-related parameters across different categories in terms of RR

Figure 4 represents the RR using a fixed effects model and 95% CI across different categories of impact of resveratrol on oral neoplastic parameters. We first explored the effect of resveratrol on oral neoplastic proliferation. This category included studies by Berta et al. [9], Masuelli et al. [10], Pradhan et al. [11], Shrotriya et al. [12], and Uzawa et al. [13]. The pooled data from these studies involved a total of 311 events in both the control and resveratrol-treated groups. The analysis revealed a summary relative risk (RR) estimate of 0.86, with a corresponding 95% CI of [0.71, 1.03]. Although the overall effect did not reach statistical significance (Z = 1.62, *P* = 0.11), the results indicated a trend towards a potential reduction in oral neoplastic proliferation with resveratrol treatment. Next, we examined the impact of resveratrol on the decrease in oral neoplastic biomarkers in the category. The studies included in this analysis were Masuelli et al. [10], Pradhan et al. [11], Shrotriya et al. [12], and Uzawa et al. [13]. The combined data from these studies involved a total of 261 events in both the control and resveratrol-treated groups. The calculated RR was 0.85, with a 95% CI of [0.69, 1.04]. Similar to the first category, the effect did not reach statistical significance (Z = 1.61, *P* = 0.11), but it suggested a potential trend towards a decrease in oral neoplastic biomarkers with resveratrol treatment. Combining all the data from both categories, which included a total of 572 events in both the control and resveratrol-treated groups, the overall impact of resveratrol on oral neoplastic parameters demonstrated an RR of 0.85, with a 95% CI of [0.74, 0.98]. This combined analysis yielded a statistically significant effect (Z = 2.28, *P* = 0.02), suggesting that resveratrol may have a role in reducing oral neoplastic parameters. Heterogeneity tests were conducted to assess the consistency of the results among the studies. For both categories and the overall analysis, low heterogeneity was observed with I^2^ values of 0%, indicating minimal variability between the studies. Moreover, subgroup differences tests indicated no significant variations between the categories (*P* = 0.91, I^2^ = 0%).

## Discussion

The significance of this study lies in the comprehensive analysis of the impact of resveratrol on oral neoplastic parameters through a systematic review and meta-analysis. The findings from the selected studies collectively highlight the potential efficacy of resveratrol as a therapeutic agent in the management of oral cancer. The observed decrease in neoplastic proliferation and promotion of neoplastic apoptosis suggest that resveratrol may hold promise in suppressing tumor growth and inducing programmed cell death, which are critical aspects of cancer control. The meta-analysis provides valuable insights into the overall effect size of resveratrol treatment on oral neoplastic parameters, with a statistically significant ES of 0.85. This indicates a clinically meaningful reduction in neoplastic growth and a potential avenue for developing resveratrol-based therapies for oral cancer patients. Moreover, the subgroup analyses conducted in this study offer additional depth to the findings by exploring potential variations among different cellular types and exposure compounds. This information is vital for tailoring treatment strategies and understanding the contexts in which resveratrol may be most effective. The implications of this study extend to future research and clinical practice. The systematic review highlights the need for further investigations to corroborate and expand upon these promising findings. Well-controlled studies and randomized clinical trials are warranted to validate the efficacy of resveratrol and elucidate the underlying molecular mechanisms responsible for its actions. Understanding these mechanisms could pave the way for the development of more targeted and personalized treatments for oral cancer patients. In the clinical context, the potential therapeutic value of resveratrol may provide an adjunct approach to conventional treatments for oral neoplasms, such as surgery, chemotherapy, and radiotherapy. Resveratrol-based therapies could potentially enhance treatment outcomes, reduce side effects, and improve the overall quality of life for oral cancer patients. Furthermore, the findings of this study could have broader implications for cancer research beyond oral neoplasms. Resveratrol's multifaceted mechanisms of action and its ability to modulate neoplastic proliferation and apoptosis might be relevant to other cancer types as well. This opens up possibilities for investigating the potential applications of resveratrol in various malignancies, further advancing our understanding of cancer biology and therapeutics.

Analysing the assessments presented in the included articles, it becomes evident that resveratrol exhibits promising effects on various aspects of oral neoplastic parameters. The studies collectively suggest that resveratrol treatment is associated with a decrease in neoplastic proliferation, which is a fundamental characteristic of cancer growth. Additionally, resveratrol has demonstrated the potential to inhibit the emergence and development of OSCC induced by different carcinogens. Notably, the combination of resveratrol with other compounds, such as curcumin or GSE, appears to enhance its effectiveness in preventing neoplastic development, as seen in the reductions of various biomarkers associated with cancer progression. Furthermore, resveratrol has shown considerable effects on other crucial aspects of oral neoplastic development, including the regulation of ROS levels, inhibition of signaling molecules downstream of growth factor receptors, and modulation of angiogenesis and metastatic biomarkers. These findings collectively highlight resveratrol's multifaceted action mechanisms, involving the promotion of apoptosis, suppression of proliferation, and induction of autophagy, which altogether contribute to its potential therapeutic value in oral cancer prevention and treatment.

In OSCC cell strains, resveratrol inhibited the rate of cell growth in a concentration- and time-dependent manner [14]. According to cell cycle analysis, resveratrol administration raised the proportion of cells in the G2/M phase while concurrently decreasing the G1 phase in a gradual way [15–18]. According to a recent investigation, resveratrol successfully inhibited cellular development and encouraged the accumulation of G1-phase cells [19]. Resveratrol demonstrated suppression of particular proliferative genes, mechanistically [20], pointing to a potential role for the compound in modifying cellular processes involved in chromatin stability. Additionally, resveratrol administration reduced the viability of CSCs cells in a dose-dependent fashion, demonstrating its deleterious impact on oral cancer precursor cells [21]. Resveratrol has been shown to regulate multiple cellular markers linked to neoplastic origin and resistance in addition to its effects on cell proliferation. Resveratrol decreased ALDH1 activity and CD44 expression in oral CSCs in different concentrations, as shown by Hu et al. [22]. Additionally, H-357-CSCs cells were treated with resveratrol [23]. These results are significant because ALDH1 and CD44 are recognised as possible indicators of radiation and/or chemotherapy resistance [24, 25]. In oral cancer cells, resveratrol administration also caused alterations in the expression of proteins that are mesenchymal-like, as well as epithelial, indicating a potential involvement in influencing the epithelial-mesenchymal transition [24].

Resveratrol also had an inhibitory effect on the adhesion, migration, and invasion of oral cancer cells. Oral cancer cell adhesion was decreased in response to resveratrol exposure at various doses [25, 26]. With higher concentrations of resveratrol, the inhibitory effects on cell motility, migration, and invasion became more pronounced [27]. These observations supported the idea that resveratrol exhibits inhibitory effects on oral cancer cell migration and invasion in vitro [28], which was supported by results published in another study [29]. Several studies examined the expression of neoplastic pathways to acquire a better understanding of the underlying mechanisms of resveratrol's effects on oral cancer cells [28]. As one article observed, while p38 phosphorylation remained constant after resveratrol therapy, ERK and JNK phosphorylation were suppressed [24–27, 29]. In studies on metalloproteinases, resveratrol significantly reduced MMP-2 activity (although MMP-9 action was unaffected) [28]. The MMP-9 enzyme activity was, however, reduced by resveratrol in a gradual range of doses [25]. According to the findings, resveratrol significantly reduces MMP-9 expression in SCC-9 cells [28]. Resveratrol recently was found to lower MMP-9 and MMP-2 levels in CSCs cells, according to one paper included in our review [11]. The findings of another paper revealed that SCC15 and SCC25 cells treated with the same quantity of resveratrol had very few condensed nuclei, but CAL27 cells displayed compacted and fragmentary foci [24, 26, 28].

This study has several limitations that should be considered in the context of the findings obtained and its future implications. Firstly, the meta-analysis was based on a relatively small number of eligible studies, which may limit the generalizability of the results. The inclusion of only five studies might not fully capture the heterogeneity of resveratrol's effects on different cellular types and exposure compounds in animal models of oral cancer. Consequently, caution should be exercised in extrapolating these findings to a broader population. Secondly, despite efforts to follow the PRISMA guidelines, the potential for publication bias cannot be entirely ruled out [30–41]. The inclusion of published studies may be biased towards those with significant results, leading to an overestimation of the observed effect size. This bias might influence the overall interpretation of resveratrol's impact on oral neoplastic parameters. Another limitation pertains to the variations in the experimental designs and assessment protocols among the included studies. Differences in the exposure concentrations, administration routes, and assessment periods may contribute to the observed heterogeneity in the meta-analysis. The lack of standardization across studies may confound the overall interpretation of the results and warrant cautious consideration. Furthermore, while the meta-analysis provides valuable insights into the collective impact of resveratrol on neoplastic proliferation and apoptosis, it does not account for potential confounding factors and individual patient characteristics. Additionally, considering the potential publication bias, efforts should be made to include unpublished data and gray literature to ensure a comprehensive and unbiased evaluation of resveratrol's impact on oral cancer. Collaborative efforts among researchers and data sharing initiatives could enhance the pool of eligible studies and increase the statistical power of future meta-analyses.

## Conclusions

The qualitative and quantitative analysis of the selected studies collectively demonstrate that resveratrol treatment is associated with a statistically significant decrease in neoplastic proliferation and a promotion of neoplastic apoptosis. These observed effects highlight the potential efficacy of resveratrol as a promising therapeutic agent in the management of oral cancer. The meta-analysis revealed an overall ES of 0.85, indicating a clinically meaningful reduction in neoplastic growth with resveratrol treatment. Subgroup analyses further explored potential variations among different cellular types and exposure compounds, providing additional depth to the findings and offering insights into resveratrol's effectiveness in specific contexts. The significance of these findings lies in the potential implications for future research and clinical practice. Resveratrol-based therapies hold promise as a complementary approach to conventional treatments for oral neoplasms. Further well-controlled studies and clinical trials are warranted to validate the observed effects and elucidate the underlying molecular mechanisms responsible for resveratrol's actions in oral cancer cells. Moreover, these assessments may extend beyond oral cancer, as resveratrol's multifaceted mechanisms of action could have broader implications in other cancer types. The study contributes to the growing body of knowledge on resveratrol's potential applications in cancer biology and therapeutics, opening up possibilities for investigating its efficacy in various malignancies.

## Data Availability

The corresponding author will have access to the data that were the basis for this article.
